# Safety and tolerability of hydroxychloroquine in health care workers and first responders for the prevention of COVID-19: WHIP COVID-19 Study

**DOI:** 10.1016/j.ijid.2021.12.343

**Published:** 2022-03

**Authors:** John E. McKinnon, Dee Dee Wang, Marcus Zervos, Matt Saval, Laurie Marshall-Nightengale, Paul Kilgore, Pardeep Pabla, Ed Szandzik, Kathleen Maksimowicz-McKinnon, William W. O'Neill

**Affiliations:** a Infectious Disease, Henry Ford Hospital, Detroit, Michigan, USA; b Division of Cardiovascular Disease, Center for Structural Heart, Henry Ford Hospital, Detroit, Michigan, USA; c Public Health Sciences, Henry Ford Hospital, Detroit, Michigan, USA; d Pharmacy, Henry Ford Hospital, Detroit, Michigan, USA; e Rheumatology, Henry Ford Hospital, Detroit, Michigan, USA

**Keywords:** Hydroxychloroquine, COVID-19, Chemoprophylaxis, Randomized trial, Health care workers

## Abstract

**Background:**

Health care workers (HCW) are among the highest risk groups for acquisition of COVID-19 because of occupational exposures. The WHIP COVID-19 Study aimed to evaluate the safety and efficacy of hydroxychloroquine (HCQ) as chemoprophylaxis for SARS-CoV-2 infection in this population.

**Methods:**

HCW, first responders, and other occupationally high-risk participants were enrolled in a randomized, placebo-controlled clinical study of HCQ from April to October 2020. The trial compared daily versus weekly HCQ with placebo and with a prospective cohort on HCQ for autoimmune diseases. Participants were followed for 8 weeks. Serology or a positive polymerase chain reaction test was used to determine laboratory confirmed clinical cases.

**Results:**

A total of 624 participants were randomized to placebo (n = 200), weekly HCQ (n = 201), daily HCQ (n = 197). For the primary safety end point, 279 (44.7%) participants experienced adverse event (AE) level II or lower (total AEs n = 589), similar rates in all randomized groups (*P* = .188) with no hospitalizations or interventions required. Only 4 laboratory confirmed COVID-19 cases occurred, with 2 in the placebo arm and one in each HCQ randomized arm.

**Conclusions:**

This randomized placebo-controlled trial was able to demonstrate the safety of HCQ outpatient chemoprophylaxis in high-risk groups against COVID-19. Future studies of chemoprophylaxis for SARS-CoV-2 are needed as the epidemic continues worldwide.

## Highlights

• Hydroxychloroquine chemoprophylaxis is safe in high-risk populations for COVID-19.
• No increased cardiovascular risks were observed with hydroxychloroquine chemoprophylaxis.
• Adverse events were similar between placebo and hydroxychloroquine treatment arms.

## Introduction

In December 2019, a novel disease caused by a new virus now known as SARS-CoV-2, commonly referred to as COVID-19, was identified in Wuhan, China (Huang et al, 2020). Since then, this deadly pandemic infection has spread worldwide (with more than 261 million cases and more than 5 million deaths as of November 2021), with observed case-fatality ratios ranging between 0.9% to 7.9% (Johns Hopkins Coronavirus Resource Center, https://coronavirus.jhu.edu/map.html) (Johns Hopkins University of Medicine, 2021).

HCW have a 3-fold increased risk of testing positive for COVID-19 compared with the general population (Nguyen et al, 2020). Disease prevention (pre-exposure [PrEP] or post-exposure [PEP]) prophylaxis and early treatment of other illnesses have been demonstrated to prevent or diminish hospitalization rates and avoid disease complications and multisystem-severe disease, and are distinct from inpatient therapeutic management (Bariola et al, 2021, Dronavalli et al, 2020, Hiba et al, 2011, Taylor et al, 2021). Transitioning from the current established procedure of clinical management of COVID-19 from a hospital-based doctrine to a community-based approach that involves outpatient chemoprophylaxis and early treatment will be a key means to prevent severe disease, avoid hospitalizations, and decrease COVID-associated morbidity and mortality. Since 1969, HCQ (and chloroquine) has been well documented to have in-vitro antiviral activity (Inglot, 1969). Antiviral activity against SARS-CoV and other viruses with chloroquine was identified in 2004 and confirmed in other in-vitro studies (Keyaerts et al, 2004, Sinha and Balayla, 2020). HCQ changes the pH at the surface of the cell membrane, affects endocytosis, inhibits aspects of nucleic acid replication, and can interfere with the glycosylation of the ACE2 receptor. Additionally, some viral proteins/enzymes can be affected by HCQ, which may affect phosphorylation of p38 mitogen-activated protein kinase and broadly impair virus assembly, restricting the new virus particle transport, release, and other key processes (Perricone et al, 2020). In Dengue virus models, HCQ activates the innate immune signaling pathways of interferon beta, activator protein 1, and nuclear factor kappa B, and induces cellular production of reactive oxygen species as host immune defense against viral infection (Wang et al, 2015). Both HCQ and chloroquine (CQ) are capable of binding to the human ACE-2 protein that serves as the CoV-2 viral receptor, and interfere with the viral S protein's ability to bind to gangliosides. Initial studies on SARS-CoV-2 showed improved HCQ activity over CQ in-vitro with lower EC50 values for HCQ (Liu et al, 2020, Wang et al, 2020).

Based on the available in-vitro and clinical data, HCQ was selected as chemoprophylaxis for persons at high risk for exposure to infected populations through their work environment, including HCW and persons employed in other high-risk occupations in our study.

## Methods

### Trial design

The study “Will Hydroxychloroquine Impede or Prevent COVID-19” (WHIP COVID-19 Study) was designed as a 3000-participant study of HCW, first responders and correctional/law officers (FR), nursing home workers (NHW), medical students (MS), public transit workers, and household family members of HCW in Michigan and Ohio. Eligible participants who were asymptomatic for prespecified signs and symptoms suggestive of COVID-19 infection were entered into the study.

The study was a randomized, placebo-controlled, double blind study with 3 active randomized arms and a comparator HCQ Cohort on maintenance full dose therapy for autoimmune disease (AD). Participants were randomized in a 1:1:1 ratio to either oral dosing of HCQ 400 mg weekly, HCQ 200 mg daily after a loading dose of 400 mg on day 1, or placebo daily. Only the unblinded pharmacist was aware of the randomized treatment assignment. Participants were provided 8-week packets of medications as per their randomization assignment. The nonrandomized HCQ Cohort was followed for 8 weeks and underwent study procedures and remained on their medications. The trial protocol, statistical analysis plan, and the de-identified trial data have been uploaded to Vivli Center for Global Clinical Research Data repository (https://doi.org/10.25934/00007320).

### Patients and ethical statement

**Figure 1:**
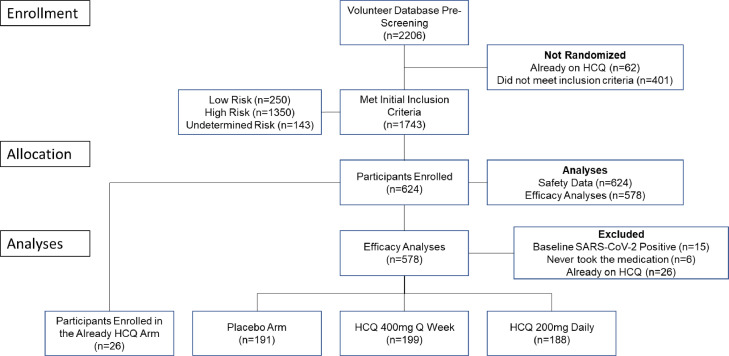
Consort flow diagram.

The study was approved by the Henry Ford Hospital (HFH) institutional review board on April 6, 2020, and the first participant was enrolled on April 10, 2020. The protocol was submitted for an investigational new drug application and received FDA approval (IND #149359), and was listed in ClinicalTrials.gov (N° NCT04341441). All participants completed an online volunteer prescreening questionnaire form, and if the participant qualified for study enrollment, an invitation to participate in the study was extended. Participants were contacted directly to complete symptom screening and informed consent process in person or electronically (consent process video and an online consent form). Verbal consent was also available for those participants not comfortable with the online process. A Data Safety Monitoring Board (DSMB) was established and reviewed the study progress and safety data monthly. The patient screening and allocation are detailed in the Consort flow diagram (Figure 1).

### Study evaluations

Participants enrolled in the study were contacted by the study staff to review study questionnaires and concomitant medications and scheduled at their preferred participating clinical site for blood draws, symptom review, and evaluation. All participants were evaluated and had laboratory evaluations done at baseline (post-enrollment), week 4 and at week 8 of the study. Weekly symptom assessments were completed via telephone and/or electronic encounters (virtual visits, e-mail), as preferred by the participant, to enhance adherence to the protocol. The weekly monitoring was designed to assess for the development of any adverse events (AEs) defined as the following: COVID-19 related symptoms, COVID-19 clinical disease, and medication adverse effects. AE reporting used the common terminology criteria for AEs, grades (1–5) and reporting based on US Department of Health and Human Services and National Institutes of Health guidelines (HHS.gov; NIH.gov).

Participants who reported symptoms suggestive of COVID-19 infection (≥2 symptoms as listed in the updated Centers for Disease Control and Prevention list for April 27, 2020, which included fever, cough, shortness of breath, chills, shaking chills, muscle pain, headache, sore throat, and new loss of smell or taste) were referred for testing and evaluation by their primary care physician or local medical center. Participants determined to be positive for COVID-19 by either study or clinical testing completed the study at that time point. Every effort was made to obtain confirmatory test results for COVID-19, including patient self-report, at study evaluations if presenting with symptoms, and from supportive medical records or study testing. Participants diagnosed with COVID-19 were asked to present for a study visit to provide a blood sample and answer an end of study questionnaire within 30 days of recovery.

Samples were collected for SARS-CoV-2 serology assessments using a SARS-CoV-2 IgG assay from Beckman Coulter, Inc, Indianapolis, Indiana, and confirmed with the Elecsys Anti-SARS-CoV-2 assay from Roche Diagnostics, Rotkreuz, Switzerland. All tests were completed following the manufacturer's specifications and were done in the HFH clinical laboratory or at the HFH Infectious Diseases Research Laboratory. Testing for COVID-19 serologies was not completed in real time because the assays were not available at the time of study initiation. Sample testing for serology was done in batches, and the initial serologic testing was completed by November 2020.

### Study outcomes

For the study primary outcome, clinical COVID-19 disease was confirmed in the participant at any time during the study if the following was determined: (a) presentation with COVID-19 symptoms during study follow-up (defined as fever ≥38° C, or ≥ non-fever symptoms that are new since baseline) and (b) laboratory confirmation of SARS-CoV-2 infection (test positive) defined as at least one positive laboratory test (reverse transcription–polymerase chain reaction [RT-PCR] and/or IgM/IgG positive serology). The laboratory test results were obtained from (a) study blood samples (IgM and IgG serology) or (b) RT-PCR test results ordered by a participant's primary care physician or local testing center or through Employee Health.

Serology results were reviewed by 2 infectious disease specialists and adjudicated as negative, positive, or unknown/indeterminate. Baseline positive serology was defined as a participant having either a positive IgG, or positive IgM serology at baseline followed by IgG seroconversion at subsequent time points.

### Statistical analyses

The study used intention to treat analysis to determine treatment efficacy based on patients who were SARS-CoV-2 negative at the baseline because the study did not have reliable assays available for COVID-19 detection at the time of initial enrollment. The primary analysis compared the rate of COVID-19 disease for each treatment group with that of the placebo group with a Mantel-Haenszel χ^2^ test with multiplicity adjustment using the Dunnett step up method. The stratifying variables were site, and high versus low-risk groups. The high-risk group included HCW (including environmental service, NHW, and MS) who worked in COVID-19 care areas, emergency rooms (ER), and intensive care units caring for COVID-19 patients and household family members. Law enforcement, FR, public transit drivers, and District Department of Transportation bus drivers were also designated as a high-risk group. Low-risk groups included HCW (including MS) who work in non–COVID-19 patient-care areas without direct patient contact or in administrative roles.

The sample size was determined with one planned interim analysis when 50% of the participants had completed their 8 weeks of treatment using an O'Brien-Fleming alpha spending method to ensure an overall type 1 error of 0.05. With a sample size of 900 per group and alpha = 0.0492, the power to detect a 32% reduction in COVID-19 disease rate (10% vs 6.8%) between the placebo group and each HCQ treated group was determined to be 87%. Based on the estimation of 5-10% of patients with SARS-CoV-2 infection present at baseline, the study required 1000 per group with a total of 3000 patients to complete the trial.

The DSMB and study chairs were provided weekly safety AE reports and convened monthly to review trial conduct and safety data. All AEs were noted, including expected and unexpected events reported by participants, and criteria for AE and severe AE (SAE) used standard definitions.

The trial had significant declines in study enrollment following the US FDA revoke of the Emergency Use Authorization (EUA) for hydroxychloroquine on June 15, 2020 (letter revoking EUA for chloroquine phosphate and hydroxychloroquine sulfate, 6/15/2020 [fda.gov]) (Food and Drug Administration, 2020). The EUA was revoked following several published articles suggesting possible increased risk for morbidity and mortality, most of which were subsequently retracted or disproven (Mehra et al, 2020, Raoult, 2020). The trial was affected by declining COVID-19 cases because of most of the enrollment occurring after the initial wave of COVID-19 had declined in Michigan and the aggressive implementation of masking and safety measures in the hospital system to prevent viral spread within our HCW population (Wang et al, 2021). The DSMB board met in November 2020 to evaluate the clinical trial for safety and possibility of completion given the interim results and study enrollment. The DSMB determined the trial study doses were safe, but because of low enrollment and low event numbers, it was recommended to be stopped early because the study end points would not be met. The WHIP COVID-19 Study was terminated on December 14, 2020.

## Results

### Study participants

All 624 enrolled participants were used for the safety analyses, and 578 participants had sufficient data for the efficacy analyses. Of note, 200 participants were randomized to the placebo arm, 201 to the 400 mg HCQ weekly dose arm, and 197 to the 200 mg HCQ daily dose arm, and 26 participants were enrolled who were taking HCQ for their AD treatment in the nonrandomized control arm. The participants were mostly female (59%), high-risk group (69%), Caucasians (85%), with a mean age of 44.9 years. Most participants were working in health care, primarily hospitals (84%) in the Detroit area, with the majority having direct patient contact (82%) and over half providing direct care to COVID-19 patients (54%) (Table 1). Sixty percent reported contact with a COVID-19 positive patient before study entry.

**Table 1:** Baseline Demographics

Variable	Response	Total (N=578)	Placebo (N=191)	400mg weekly (N=199)	200mg Daily (N=188)
Risk group	Lower Risk	160 (28%)	55 (29%)	56 (8%)	49 (26%)
	High Risk	418 (72%)	136 (71%)	143 (72%)	139 (74%)
Gender	F	336 (58%)	114 (60%)	108 (54%)	114 (61%)
	M	242 (42%)	77 (40%)	91 (46%)	74 (39%)
Age in years	Mean (SD)	44.9 (11.9)	44.1 (12.7)	45.7 (11.6)	44.9 (11.4)
Race	White	495 (86%)	161 (84%)	177 (89%)	157 (84%)
	Black	24 (4%)	9 (5%)	5 (3%)	10 (5%)
	AS/IN/PI	38 (7%)	15 (8%)	10 (5%)	13 (7%)
	Unknown	21 (4%)	6 (3%)	7 (4%)	8 (4%)
Recent history of travel outside of Michigan?	Yes	124 (22%)	38 (20%)	50 (25%)	36 (19%)
	No	449 (78%)	150 (80%)	148 (75%)	151 (81%)
Exposed to anyone diagnosed with COVID19 confirmed by laboratory	Yes	348 (60%)	115 (60%)	116 (59%)	117 (62%)
	No	228 (40%)	76 (40%)	81 (41%)	71 (38%)
Employment	Hospital System	486 (84%)	163 (86%)	163 (83%)	155 (83%)
	First Responders	9 (2%)	4 (2%)	3 (2%)	2 (1%)
	Skilled Nursing/Rehab Facility	1 (0%)	0 (0%)	1 (0%)	0 (0%)
	Other	82 (14%)	23 (12%)	29 (15%)	30 (16%)
Do you have direct contact with patients?	Yes	472 (82%)	159 (83%)	159 (80%)	154 (82%)
	No	98 (17%)	30 (16%)	35 (18%)	33 (18%)
	NA	8 (1%)	2 (1%)	5 (3%)	1 (1%)
Do you work in an area categorized as direct COVID- 19 Care?	Yes	315 (54%)	105 (55%)	101 (51%)	109 (58%)
	No	245 (42%)	80 (42%)	88 (44%)	77 (41%)
	NA	18 (3%)	6 (3%)	10 (5%)	2 (1%)

### Safety and adverse events

Of the 624 participants enrolled, only 279 (44.7%) experienced an AE, with a total AEs reported during the study at 589 events. All study AEs were level 1 or 2 in severity. No AE grade 3 or 4, or SAEs or visits to the ER or hospital occurred during the study (Table 2).

**Table 2:** Adverse Events by Treatment Arm

Adverse Event	Treatment Group																			
	Total				Placebo				400mg HCQ weekly				200mg HCQ Daily				HCQ Cohort			
	Grade			All	Grade			All	Grade			All	Grade			All	Grade			All
														
	1	2	3 & 4		1	2	3 & 4		1	2	3 & 4		1	2	3 & 4		1	2	3 & 4	
Gastrointestinal disorders	148	1	0	149	52	0	0	52	42	1	0	43	54	0	0	54	0	0	0	0
Nervous system disorders	113	5	0	118	41	1	0	42	31	2	0	33	41	2	0	43	0	0	0	0
Respiratory, thoracic and mediastinal disorders	62	3	0	65	20	1	0	21	26	1	0	27	16	1	0	17	0	0	0	0
General disorders and administration site conditions	37	4	0	41	15	2	0	17	13	2	0	15	9	0	0	9	0	0	0	0
Cardiac disorders	37	6	0	43	9	1	0	10	14	2	0	16	14	3	0	17	0	0	0	0
Musculoskeletal and connective tissue disorders	37	10	0	47	15	1	0	16	12	5	0	17	10	3	0	13	0	1	0	1
Psychiatric disorders	24	2	0	26	7	0	0	7	11	2	0	13	6	0	0	6	0	0	0	0
Skin and subcutaneous tissue disorders	22	3	0	25	3	0	0	3	8	2	0	10	11	1	0	12	0	0	0	0
Ear and labyrinth disorders	23	4	0	27	7	1	0	8	8	1	0	9	8	2	0	10	0	0	0	0
Eye disorders	15	1	0	16	6	0	0	6	1	0	0	1	8	1	0	9	0	0	0	0
All	531	58	0	589	179	9	0	188	169	24	0	193	183	23	0	206	0	2	0	2

Note: No Serious Adverse Events were recorded in any of the treatment groups. No Grade 3 or 4 adverse events were recorded in any of the treatment groups. Top 10 reported adverse event groups are listed above. All other AE disorders were less than 10 events total per group.

Grade 1 and 2 AEs in participants were equally distributed between the randomized groups, with 85 participants in the placebo arm, 95 in the weekly HCQ arm, and 97 in the daily HCQ arm (*P* = .38). The patients on full dose HCQ only reported 2 participants with AEs. The severity of the events was similar per randomized group between grade 1 and 2 (*P* = .188).

The most common adverse effect reported was gastrointestinal (GI) disorders (eg, nausea and gastrointestinal upset). GI symptoms were similarly distributed between randomized treatment arms, with 52 AEs in the placebo arm, and 42 in HCQ weekly and 54 in the HCQ daily arm, respectively. Most AEs were grade 1. Similarly, nervous system disorders (primarily headaches) were equally distributed between groups. Cardiac disorders were only palpitations, mostly grade 1, without any patient requiring referral to the ER or hospital. No statistically significant differences in AEs distribution between groups were identified during the study at any time point.

### Clinical COVID-19 cases

During the clinical trial, 35 participants were considered unknown/indeterminate for COVID-19 status because of serologies not confirming positive seroconversion, clinical symptoms without a positive serology or PCR test, or missing laboratories or visits to confirm COVID-19 infection. All patients suspected of COVID-19 infection were referred to their Employee Health or primary care providers for further evaluation and testing, and none required hospitalization. Four patients were confirmed positive by laboratory results (PCR or serology) during the study, with 3 participants also demonstrating clinical disease, which was the study primary end point (Table 3). The placebo arm had 2 patients with positive laboratory testing, with 1 with confirmed disease. The two randomized HCQ arms had one confirmed positive COVID-19 case each. No cases of COVID-19 or positive serologies were seen in the 25 patients who were chronically on HCQ. No statistically significant difference was seen between groups for positive serologies or confirmed COVID-19 cases because of low event numbers (Table 4). Multiple imputation analyses also demonstrated *P* values between .27 to .85 for COVID-19 test positive results and .69 to 1.0 for confirmed COVID-19 disease analyses.

**Table 3:** COVID-19 Serology and Clinical Results

		Treatment Group						Total	
		Placebo		400mg HCQ weekly		200mg HCQ Daily			
		N	%	N	%	N	%	N	%
Covid Test*	Unk/Ind^†^	13	6.8	12	6.03	10	5.31	35	6.05
	Negative	176	92.14	186	93.46	177	94.14	539	93.25
	Positive	2	1.04	1	0.5	1	0.53	4	0.69
Covid Disease**	Unk/Ind	13	6.8	12	6.03	10	5.31	35	6.05
	Negative	177	92.67	186	93.46	177	94.14	540	93.42
	Positive	1	0.52	1	0.5	1	0.53	3	0.51

Note: *p-value=0.699 for Covid Test positive; **p-value=1.0 for Covid disease positive using Fisher exact test, on observed data assuming missing at random. ^†^UNK/Ind refers to unknown/indeterminate serology/laboratory results for COVID-19 infection as some participants did not complete all laboratory assessments or the serological determination of COVID-19 could not be done due to inconclusive test results.

**Table 4:** COVID-19 Infections in participants will full laboratory data in both randomized and non-randomized groups.

COVID-19 Diagnosis		Treatment Group							
		Placebo		400mg HCQ weekly		200mg HCQ Daily		HCQ AD Therapy^†^	
		N	%	N	%	N	%	N	%
Infection Status (test)*	Negative	189	99	198	99.5	186	98.9	25	100
	Positive	1	0.52	0	0	0	0	0	0
Clinical Disease**	Positive	1	0.52	1	0.5	2	1.06	0	0

Note: *Patients only testing positive for COVID-19 serology were included. **Participants with both serology positive for COVID-19 infection and clinical symptoms consistent with COVID-19 disease were included. P-value for the comparison between groups including the participants with **^†^**HCQ therapy for autoimmune diseases was 0.75.

## Discussion

In our randomized, placebo-controlled trial for HCQ chemoprophylaxis using either daily or weekly dosing, we were able to demonstrate the safety of the HCQ outpatient regimen compared with placebo. No AEs grade 3 or 4 occurred during the study; no patient required ER visits or hospitalization for adverse effects of the medication, and most of the AEs documented in the study follow-up of 624 patients were grade 1. This study clearly demonstrates the known safety profile of HCQ, which is consistent with numerous studies of rheumatologic use of daily HCQ chronically for disease treatment (Fram et al, 2020). Unfortunately, despite its established safety profile since 1949, this long track record of safety and tolerability was questioned based on retrospective chart review studies with confounding patient populations in the early phase of the pandemic, at a time when prompt testing was not available and treatment was delayed, or other instances of misleading publications (Magagnoli et al, 2020, Mehra et al, 2020, Rosenberg et al, 2020). The safety of outpatient HCQ has been demonstrated in multiple previous studies in several different conditions, including the COVID-19 global pandemic (Observational Health Data Sciences and Informatics (OHDSI), 2020, Perricone et al, 2020). A major concern expressed by the FDA in revoking the EUA approval for HCQ was the potential for cardiac arrhythmias due to possible QTc prolongation. No patient in our study developed a cardiac arrhythmia and/or required medical evaluation for cardiac symptoms. The COVID-19 cardiac and vascular endothelial mechanisms of dysfunction are now better understood, and the associated risk for developing cardiac arrhythmias has been deemed due to viral myocarditis (Douedi et al, 2021, Nabeh et al, 2021). Myocarditis, cardiac arrhythmias, and cardiomyopathy are known to be associated with QTc prolongation, and hence, early scientific publications’ association of HCQ use with QTc prolongation in the late inpatient and critical care setting may have suffered from lack of early scientific understanding of the pathophysiology of COVID-19 (Boehmer et al, 2021, Onohuean et al, 2021). This finding has been supported by several other studies, including an Oxford study that examined cardiac arrhythmia outcomes and obtained for its random effects meta-analysis result, RR = 1.08, *P* value = .36 for HCQ + azithromycin (AZ) use versus HCQ + amoxicillin use (another broad-spectrum antibiotic), with the fixed-effects meta-analysis demonstrating a RR = 1.04, *P* value = .41. The study clearly demonstrated that cardiac arrhythmia AEs are not appreciably increased by combining HCQ with AZ (Observational Health Data Sciences and Informatics [OHDSI], 2020). HCQ was compared with sulfasalazine use, with no difference in cardiac arrhythmia risk for HCQ, with a slightly lower RR = 0.89, *P* value = .13. Another review of published cardiac complications attributed to HCQ in the pre–COVID-19 era identified only 69 articles where most cardiotoxicity events were reversible with standard of care and only 2 fatalities were identified, and both were in acute intentional overdoses (Fram et al, 2020). Other concerns for hemolysis and methemoglobinemia with HCQ have not been reported in large clinical prophylaxis trials.

More than 70 US and international studies and trials for HCQ PrEP and PEP have been published since the initiation of this protocol (Monti et al, 2020). Boulware and colleagues published a trial evaluating the benefit of HCQ as a PEP regimen. The trial did not demonstrate any significant benefit, but it was acknowledged that there were flaws with the design of the study and that further research was needed. This study also did not demonstrate any increases in cardiovascular or SAE or mortality in the HCQ treatment arm (Lofgren et al, 2020). Other trials were done with randomized samples, including studies by Skipper and colleagues, Rajasingham and colleagues, and Mitja and colleagues (Boulware et al, 2020, Mitjà et al, 2021, Rajasingham et al, 2021, Skipper et al, 2020). A trial from Spain similar to our study demonstrated similar safety findings and a trend toward HCQ benefit in prevention of COVID-19 in HCW but was unable to reach statistical significance because of difficulty in enrolling patients secondary to negative reports regarding HCQ therapy (Rojas-Serrano et al, 2021). Unfortunately, the safety profile of HCQ shown in multiple trials is not reflected in the current guidelines from the WHO, which are based on only 6 studies solely from North America and Europe (World Health Organization, 2021). All these studies suffered from different limitations, including early termination, delayed intervention, underpowered sample sizes owing to missed accrual targets, and inability to provide precise estimates of efficacy of the HCQ strategy while showing numerical benefit for HCQ strategies. A recently published trial by Seet and colleagues used a cluster randomization strategy of 3037 men to evaluate several prophylaxis strategies in a well-controlled setting in Singapore. The study demonstrated absolute risk reductions for laboratory confirmed COVID-19 infection for oral HCQ (21%, 2%–42%) and for povidone-iodine throat spray (23%, 7%–39%) over vitamin C control with a corrected alpha <0.0125 (Seet et al, 2021). In this large study, HCQ did not affect QTc interval in treated patients. Ivermectin and zinc did not show a benefit in this study over vitamin C. Several meta-analyses have been done to address these limitations, demonstrating efficacy of HCQ in the outpatient setting when evaluating the randomized clinical trial data. Ladapo and colleagues, using fixed-effects and random-effects calculations, were able to show a 24% reduced outcome risk for the composite outcome of COVID-19 infection, hospitalization, and death (*P* = .025) for the HCQ intervention (Ladapo et al, 2020). Similarly, a meta-analysis by Million and colleagues of 20 available reports including 105,040 patients demonstrated that chloroquine and its derivatives improve clinical and biological outcomes and reduce mortality by a factor of three in COVID-19 patients (Million et al, 2020). In a recent Indian HCW retrospective PrEP cohort study of 12,089 participants funded by the Indian Council of Medical Research, the use of HCQ prophylaxis was associated with declines in COVID-19 positivity from 34% up to 72%, depending on the frequency of HCQ use in adjusted OR, with no difference in hospitalization rates (Badyal Dinesh et al, 2021). Dev and colleagues found that sanitation workers and technicians at the hospitals were at higher risk for COVID-19 infection. This correlated with inappropriate use of PPE and lack of use of HCQ. In participants using HCQ, the risk reduction was 26% (RR 0.74, *P* .003) in 260 participants on treatment versus 499 controls (Dev et al, 2021). A recent HCQ treatment review using the Cochrane review manager identified 19 treatment trials out of 903 studies screened. The analyses demonstrated significant benefits in both improved rates of virologic cure (OR = 2.08) and of radiological cure (OR = 3.89), but no effect on mortality (aHR = 1.05) in the symptomatic treatment settings (Mittal et al, 2021).

The WHIP COVID-19 Study was not able to demonstrate efficacy of the HCQ strategy because only 4 confirmed cases of COVID-19 were identified in the study, a key limitation to assess the strategy efficacy. The low event rate was due to previously mentioned concomitant factors that occurred during enrollment. Our study participants’ risk was significantly decreased in part by very aggressive masking and social distancing interventions initiated at our facilities early in the epidemic, impacting the positivity rate for COVID-19 in the HFH System, from which over 60% of our participant pool was derived. The interventions were considered to be highly effective (Wang et al, 2021). The pandemic rates also declined in the state during the most active period of recruitment, and therefore, the community exposure rates declined during the period of April to October of 2020 (US Department of Health and Human Services, 2021). Participant acceptance of HCQ also declined with the withdrawal of the EUA approval.

However, the study was able to demonstrate conclusively that no increased risk for AEs was seen between the HCQ treatment arms as compared with placebo. Because of limitations with study participants presenting in person to the research or hospital settings during the pandemic, the participants could not be followed on site, and COVID-19 cases that may have been detected with more active in-person follow-up and with real-time testing could have been missed. All symptomatic patients were referred for testing and evaluation because of symptoms but infrequently followed up for these assessments, whereas none required hospitalization. Another limitation was lack of availability of accurate real-time assays to detect COVID-19 during the early period of the pandemic. Both assay availability and resource allocation of the available tests for clinical use prohibited potential real time testing of patients and case identification.

In summary, the WHIP COVID-19 Study was able to confirm that HCQ when administered in the outpatient setting for occupationally high-risk groups for COVID-19 infection is safe as either a daily or weekly dose. Meta-analyses and international studies have shown the value and safety of HCQ as a chemoprophylactic strategy. With the emergence of multiple SARS-CoV-2 variants and diminished effectiveness of currently available vaccines, chemoprophylaxis should be more fully evaluated as part of a comprehensive strategy to identify effective and safe regimens and interventions to be made available as new variants emerge and especially for the vulnerable populations for whom vaccine antibody response and protection will likely be weak or ineffective (McCullough et al, 2020, Pegu et al, 2021, Singh et al, 2021, Tenforde et al, 2021, Tregoning et al, 2021).

## Financial support

This study was supported by internal funding from Henry Ford Health System, the Bill and Melinda Gates Foundation (Investment ID: INV-018560), and multiple philanthropic donations from donors in Michigan and the United States that supported the goal of COVID-19 prevention studies.

## Declaration of Competing Interest

None of the investigators have potential conflict of interest related to this article.

## Appendix Supplementary materials

Image, application 1

## References

[bib0001] Dinesh Badyal, Sujith J Chandy, Kaur Chugh Preeta, Atiya Faruqui, Y G, Avijit Hazra, Sandhya Kamat (2021). Hydroxychloroquine for SARS CoV2 Prophylaxis in Healthcare Workers – A Multicentric Cohort Study Assessing Effectiveness and Safety. Journal of The Association of Physicians of India.

[bib0002] Bariola JR, McCreary EK, Wadas RJ, Kip KE, Marroquin OC, Minnier T (2021). Impact of Bamlanivimab Monoclonal Antibody Treatment on Hospitalization and Mortality Among Nonhospitalized Adults With Severe Acute Respiratory Syndrome Coronavirus 2 Infection. Open forum infectious diseases.

[bib0003] Boehmer TK, Kompaniyets L, Lavery AM, Hsu J, Ko JY, Yusuf H (2021). Association Between COVID-19 and Myocarditis Using Hospital-Based Administrative Data - United States, March 2020-January 2021. MMWR Morbidity and mortality weekly report.

[bib0004] Boulware DR, Pullen MF, Bangdiwala AS, Pastick KA, Lofgren SM, Okafor EC (2020). A Randomized Trial of Hydroxychloroquine as Postexposure Prophylaxis for Covid-19. The New England journal of medicine.

[bib0005] Dev N, Meena RC, Gupta DK, Gupta N, Sankar J. (2021). Risk factors and frequency of COVID-19 among healthcare workers at a tertiary care centre in India: a case-control study. Transactions of the Royal Society of Tropical Medicine and Hygiene.

[bib0006] Douedi S, Mararenko A, Alshami A, Al-Azzawi M, Ajam F, Patel S (2021). COVID-19 induced bradyarrhythmia and relative bradycardia: An overview. Journal of arrhythmia.

[bib0007] Dronavalli M, Lord H, Alexander K, Boonwaat L, Pal N, Fletcher-Lartey SM. (2020). Effectiveness of Oseltamivir Prophylaxis in Influenza Outbreaks in Residential Aged Care. Journal of epidemiology and global health.

[bib0008] Food and Drug Administration. Food and Drug Administration (FDA) revoke the Emergency Use Autorization (EUA) for emergency use of oral formulations of chloroquine phosphate (CQ) and hydroxychloroquine sulfate (HCQ). https://www.fda.gov/media/138945/download 2020.

[bib0009] Fram G, Wang DD, Malette K, Villablanca P, Kang G, So K, et al. Cardiac Complications Attributed to Hydroxychloroquine: A systematic review of the Literature Pre-COVID-19. Submitted 2020. 10.2174/1573403X16666201014144022 PMC8640856 33059567

[bib0010] Hiba V, Chowers M, Levi-Vinograd I, Rubinovitch B, Leibovici L, Paul M. (2011). Benefit of early treatment with oseltamivir in hospitalized patients with documented 2009 influenza A (H1N1): retrospective cohort study. The Journal of antimicrobial chemotherapy.

[bib0011] Huang C, Wang Y, Li X, Ren L, Zhao J, Hu Y (2020). Clinical features of patients infected with 2019 novel coronavirus in Wuhan, China. Lancet.

[bib0012] Inglot A. (1969). Comparison of the antiviral activity in vitro of some non-steroidal anti-inflammatory drugs. Journal of General Virology.

[bib0013] Johns Hopkins University of Medicine. Coronavirus Resource Center. https://coronavirus.jhu.edu/data/mortality; 2021. [Accessed August 20 2021].

[bib0014] Keyaerts E, Vijgen L, Maes P, Neyts J, Ranst MV. (2004). In vitro inhibition of severe acute respiratory syndrome coronavirus by chloroquine. Biochemical and Biophysical Research Communications.

[bib0015] Ladapo JA, McKinnon JE, McCullough PA, Risch HA. (2020). Randomized Controlled Trials of Early Ambulatory Hydroxychloroquine in the Prevention of COVID-19 Infection, Hospitalization, and Death: Meta-Analysis. medRxiv.

[bib0016] Liu J, Cao R, Xu M, Wang X, Zhang H, Hu H (2020). Hydroxychloroquine, a less toxic derivative of chloroquine, is effective in inhibiting SARS-CoV-2 infection in vitro. Cell Discov.

[bib0017] Lofgren SM, Nicol MR, Bangdiwala AS, Pastick KA, Okafor EC, Skipper CP (2020). Safety of Hydroxychloroquine among Outpatient Clinical Trial Participants for COVID-19. medRxiv.

[bib0018] Magagnoli J, Narendran S, Pereira F, Cummings T, Hardin JW, Sutton SS (2020). Outcomes of hydroxychloroquine usage in United States veterans hospitalized with Covid-19. medRxiv.

[bib0019] McCullough PA, Alexander PE, Armstrong R, Arvinte C, Bain AF, Bartlett RP (2020). Multifaceted highly targeted sequential multidrug treatment of early ambulatory high-risk SARS-CoV-2 infection (COVID-19). Reviews in cardiovascular medicine.

[bib0020] Mehra MR, Ruschitzka F, Patel AN. (2020). Retraction—Hydroxychloroquine or chloroquine with or without a macrolide for treatment of COVID-19: a multinational registry analysis. The Lancet.

[bib0021] Million M, Gautret P, Colson P, Roussel Y, Dubourg G, Chabriere E (2020). Clinical Efficacy of Chloroquine derivatives in COVID-19 Infection: Comparative meta-analysis between the Big data and the real world. New Microbes and New Infections.

[bib0022] Mitjà O, Corbacho-Monné M, Ubals M, Alemany A, Suñer C, Tebé C (2021). A Cluster-Randomized Trial of Hydroxychloroquine for Prevention of Covid-19. The New England journal of medicine.

[bib0023] Mittal N, Mittal R, Gupta MC, Kaushal J, Chugh A, Khera D (2021). Systematic review and meta-analysis of efficacy and safety of hydroxychloroquine and chloroquine in the treatment of COVID-19. Journal of family medicine and primary care.

[bib0024] Monti M, Vertogen B, Masini C, Donati C, Lilli C, Zingaretti C (2020). Hydroxychloroquine as Prophylaxis for COVID-19: A Review. Frontiers in pharmacology.

[bib0025] Nabeh OA, Helaly MM, Menshawey R, Menshawey E, Nasser MMM (2021). Diaa El-Deen AM. Contemporary approach to understand and manage COVID-19-related arrhythmia. The Egyptian heart journal: (EHJ): official bulletin of the Egyptian Society of. Cardiology.

[bib0026] Nguyen LH, Drew DA, Joshi AD, Guo CG, Ma W, Mehta RS (2020). Risk of COVID-19 among frontline healthcare workers and the general community: a prospective cohort study. medRxiv.

[bib0027] Observational Health Data Sciences and Informatics (OHDSI). Oxford-led international research finds hydroxychloroquine safe in over 130,000 patients. https://sundiatapost.com/oxford-led-international-research-finds-hydroxychloroquine-safe-in-over-130000-patients/ 2020.

[bib0028] Onohuean H, Al-Kuraishy HM, Al-Gareeb AI, Qusti S, Alshammari EM, Batiha GE (2021). Covid-19 and development of heart failure: mystery and truth. Naunyn-Schmiedeberg's archives of pharmacology.

[bib0029] Pegu A, O'Connell S, Schmidt SD, O'Dell S, Talana CA, Lai L (2021). Durability of mRNA-1273 vaccine-induced antibodies against SARS-CoV-2 variants. Science (New York, NY).

[bib0030] Perricone C, Triggianese P, Bartoloni E, Cafaro G, Bonifacio AF, Bursi R (2020). The anti-viral facet of anti-rheumatic drugs: Lessons from COVID-19. Journal of Autoimmunity.

[bib0031] Rajasingham R, Bangdiwala AS, Nicol MR, Skipper CP, Pastick KA, Axelrod ML (2021). Hydroxychloroquine as Pre-exposure Prophylaxis for Coronavirus Disease 2019 (COVID-19) in Healthcare Workers: A Randomized Trial. Clinical infectious diseases: an official publication of the Infectious Diseases Society of America.

[bib0032] Raoult D. (2020). Lancet gate: a matter of fact or a matter of concern. New Microbes New Infect.

[bib0033] Rojas-Serrano J, Thirion-Romero AMP-VI, Vázquez-Pérez J, Ramírez-Venegas FM-NA, Pérez-Kawabe KM, Pérez-Padilla R (2021). Hydroxychloroquine For Prophylaxis Of COVID-19 In Health Workers: A Randomized Clinical Trial. medRxiv.

[bib0034] Rosenberg ES, Dufort EM, Udo T, Wilberschied LA, Kumar J, Tesoriero J (2020). Association of Treatment With Hydroxychloroquine or Azithromycin With In-Hospital Mortality in Patients With COVID-19 in New York State. Jama.

[bib0035] Seet RCS, Quek AML, Ooi DSQ, Sengupta S, Lakshminarasappa SR, Koo CY (2021). Positive impact of oral hydroxychloroquine and povidone-iodine throat spray for COVID-19 prophylaxis: An open-label randomized trial. International journal of infectious diseases: IJID: official publication of the International Society for Infectious Diseases.

[bib0036] Singh J, Pandit P, McArthur AG, Banerjee A, Mossman K. (2021). Evolutionary trajectory of SARS-CoV-2 and emerging variants. Virology journal.

[bib0037] Sinha N, Balayla G. (2020). Hydroxychloroquine and covid-19. Postgraduate Medical Journal.

[bib0038] Skipper CP, Pastick KA, Engen NW, Bangdiwala AS, Abassi M, Lofgren SM (2020). Hydroxychloroquine in Nonhospitalized Adults With Early COVID-19: A Randomized Trial. Annals of internal medicine.

[bib0039] Taylor PC, Adams AC, Hufford MM, de la Torre I, Winthrop K, Gottlieb RL (2021). Neutralizing monoclonal antibodies for treatment of COVID-19. Nature reviews Immunology.

[bib0040] Tenforde MW, Patel MM, Ginde AA, Douin DJ, Talbot HK, Casey JD (2021).

[bib0041] Tregoning JS, Flight KE, Higham SL, Wang Z, Pierce BF. (2021). Progress of the COVID-19 vaccine effort: viruses, vaccines and variants versus efficacy, effectiveness and escape. Nature reviews Immunology.

[bib0042] US Department of Health & Human Services. https://healthdata.gov/Community/COVID-19-State-Profile-Report-Michigan/s8hn-gz3c; 2021. [Accessed August 13, 2021.

[bib0043] Wang DD, O'Neill WW, Zervos MJ, McKinnon JE, Allard D, Alangaden GJ (2021). Association Between Implementation of a Universal Face Mask Policy for Healthcare Workers in a Health Care System and SARS-CoV-2 Positivity Testing Rate in Healthcare Workers. J Occup Environ Med.

[bib0044] Wang LF, Lin YS, Huang NC, Yu CY, Tsai WL, Chen JJ (2015). Hydroxychloroquine-inhibited dengue virus is associated with host defense machinery. Journal of interferon & cytokine research: the official journal of the International Society for Interferon and Cytokine Research.

[bib0045] Wang M, Cao R, Zhang L, Yang X, Liu J, Xu M (2020). Remdesivir and chloroquine effectively inhibit the recently emerged novel coronavirus (2019-nCoV) in vitro. Cell Res.

[bib0046] World Health Organization. WHO Living guideline: Drugs to prevent COVID-19; 2021. [Accessed December 14 2021]. 35917395

